# Supplementary material for: Portable real-time colorimetric LAMP-device for rapid quantitative detection of nucleic acids in crude samples
Source: Sci Rep. 2022 Mar 8;12:3775. doi: 10.1038/s41598-022-06632-7 (PMC8904468; doi:10.1038/s41598-022-06632-7)
Supplement: Supplementary file 1 — Supplementary Information. [file 41598_2022_6632_MOESM1_ESM.docx]

***Supplementary Material***

**Portable real-time colorimetric LAMP device for rapid quantitative detection of nucleic acids in crude samples**

**G. Papadakis^1,2 *^, A. K. Pantazis^1,2^, N. Fikas^1,2^, S. Chatziioannidou^1,2,3^, V. Tsiakalou^1^, K. Michaelidou^4^, V. Pogka^5^, M. Megariti^1^, M. Vardaki^1,3^, K. Giarentis^3^, J. Heaney^6,7^, E. Nastouli^6,7^, T. Karamitros^5^, A. Mentis^5^, A. Zafiropoulos^8^, G. Sourvinos^8^, S. Agelaki^3,9^, E. Gizeli^1,3*^**

^1^ Institute of Molecular Biology and Biotechnology, Foundation for Research and Technology-Hellas,100 N. Plastira str., Heraklion 70013, Greece

^2^ BIOPIX DNA TECHNOLOGY PC, Science and Technology Park of Crete, 100 N. Plastira str, Heraklion, 70013, Greece

^3^Department of Biology, University of Crete, Voutes, Heraklion 70013, Greece

^4^ Laboratory of Translational Oncology, School of Medicine, University of Crete, Heraklion, 71500, Greece

^5^ National SARS-CoV-2 Reference Laboratory, Hellenic Pasteur Institute, 127 Vas. Sofias Ave., Athens 11521, Greece

^6^ Advanced Pathogens Diagnostics Unit, University College London Hospitals NHS Trust, London WC1H 9AX

^7^ UCL Great Ormond Street Institute of Child Health

^8^ Laboratory of Clinical Virology, School of Medicine, University of Crete, Heraklion, 71500, Greece

^9^ Department of Medical Oncology, University General Hospital, Heraklion, 71110, Greece

*Corresponding authors: gpapadakis@biopix-t.com; [gizeli@imbb.forth.gr](mailto:gizeli@imbb.forth.gr)

# In-house developed Android application


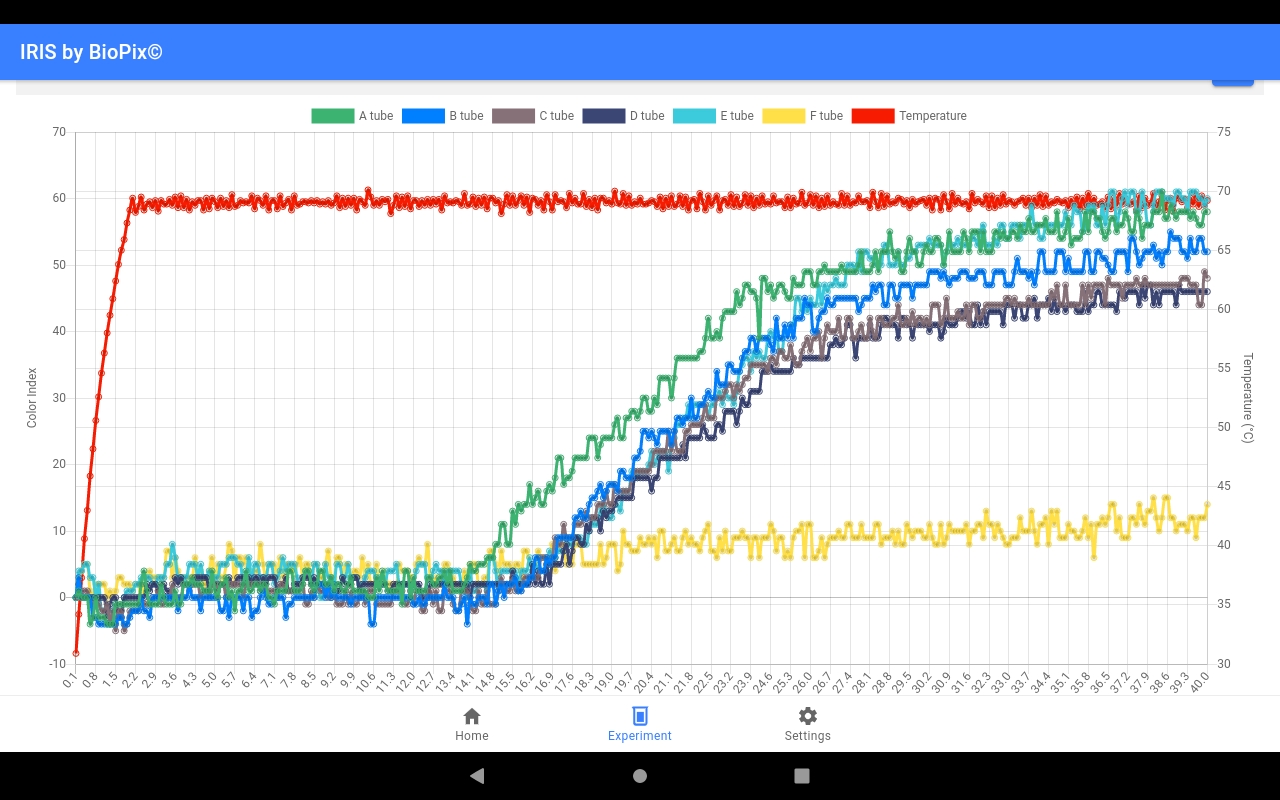


**Fig. S1**: Screen-shot of the in-house developed Android application. The settings to be adjusted include the temperature, run time, type of dye, time interval for capturing images and option for USB storage.

# Digital image analysis

**Fig. S2**: **Digital image analysis.** (a) Series of images corresponding to different pH values between pH 6 and 8 in respect to the phenol red indicator. Image source: <https://www.testallcolour.com/blog/post/what-is-phenol-red-in-swimming-pools/>. (b) Raw pixel values extracted from several images (see a) correlating different pH values to color change using the phenol red pH indicator. (c) phenol red: change in pixels as function of pH using data from figure S2b and following three formulas; Green-Blue (G-B), Green-Red (G-R) and Red-Blue (R-B). (d) Change in pixels of phenol red based LAMP reactions spiked with 0 (N) and 10^5^ (P) lysed bacteria by applying the Green-Blue formula. The first 8 minutes were omitted. Reactions took place in a pre-warmed oven at 63^o^C with a glass door that allowed video capturing with a camera placed outside the door. (e) Change in pixels of HNB based LAMP reactions spiked with 0 (N) and 10^5^ (P) bacteria by applying the Green-Blue and Green-Red formulas. With the HNB indicator, the Green-Red formula resulted in better discrimination (Fig. S2e); this could be explained by the fact that the purple to sky blue transition involved more prominent changes in the green and red than in the blue channel.

# Performance evaluation

**Fig. S3**: **LAMP speed of detection.** (a) Variation in the time point (min) in which LAMP preparations (containing the same amount of starting template but different color indicator) show a change in the slope of the real-time colorimetric curve when changing the position of the tube inside the tubes holder (see Fig. 1b). Each bar is the average of 3 replicates at 2 different slots in the holder (total of 6 measurements). (b) Comparison of the speed of detection of a LAMP reaction containing 10 bacteria as starting template using different combinations of 2 enzymes (Bst2, Bsm), 2 colorimetric indicators (HNB, phenol red) and inside 2 real time systems (qcLAMP device, BIORAD). Bst2-c: Bst2 warm start polymerase mixed with either phenol red or HNB, tested with qcLAMP; Bsm-c: Bsm polymerase (20 Units) with HNB, tested with qcLAMP; Bst2-f: Bst2 warm start with LAMP fluorescent dye tested in a real-time PCR machine. Error bars represent standard deviation of at least triplicate measurements.

**Fig. S4**: **Temperature stability of the device.** Temperature stability during operation of the qcLAMP device with a power bank and in comparison, to a standard power supply.

# qcLAMP curves for Influenza/SARS-CoV-2

**Fig S5**: **qcLAMP tests with Influenza A and SARS-CoV-2 template.** (a) Typical real time colorimetric LAMP curves for Influenza A. (b) Average time-to-positive for 21 positive samples with the same initial target concentration (10^9^ copies/reaction). (c) Real-time curves of 28 samples (21 positive, 7 negative). (d) Real time curves for 0 to 200 copies of SARS-CoV-2 synthetic RNA.

# Electronics design and smartphone app development


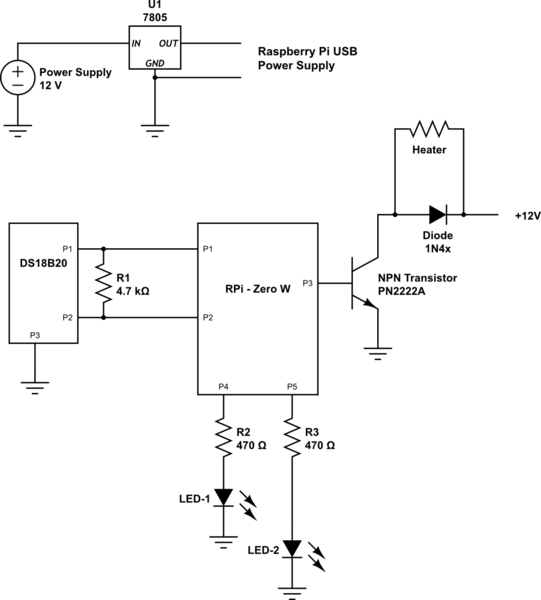


**Fig. S6**: **Electronics design.** Schematic representation of the custom PCB RPi Zero W Hat for controlling temperature sensor (DS18B20), Heating element, and LEDs.


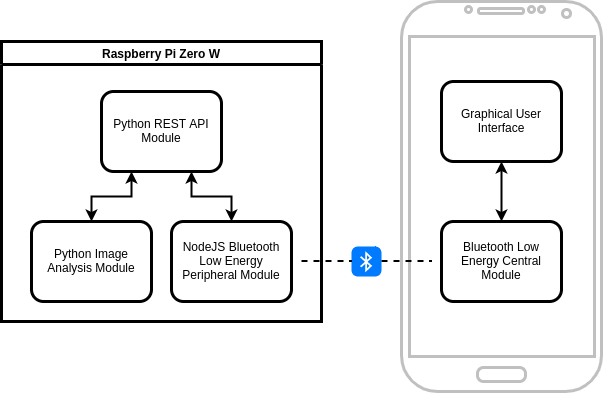


**Fig. S7**: **Systems’ architecture at software layer.** The Software layer of the device is divided into two categories: the software running on the RPi and the software deployed as an Android application on a mobile device.


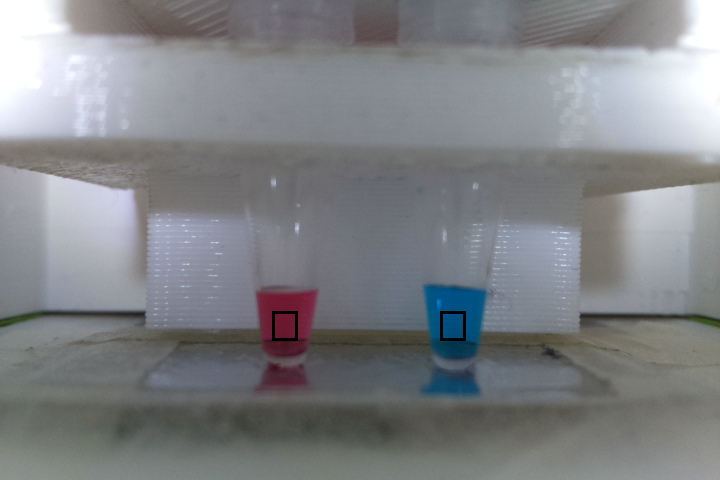


**Fig. S8**: **Snapshot of the reaction tubes in the qcLAMP device.** The application of the colorimetric analysis is performed in predefined areas depicted in the black rectangles. Photo Credit: N. Fikas Photographer Institution: IMBB-FORTH.
